# The need for the GREAT+ score to predict relapse in Graves’ disease: a questionnaire among patients and internal medicine specialists

**DOI:** 10.1007/s40618-024-02358-7

**Published:** 2024-03-25

**Authors:** H. I. Jansen, C. Heuveling van Beek, P. H. Bisschop, A. C. Heijboer, E. Bruinstroop, A. Boelen

**Affiliations:** 1 Department of Laboratory Medicine, Endocrine Laboratory, Amsterdam UMC Location Vrije Universiteit Amsterdam, Amsterdam, The Netherlands; 2 Department of Laboratory Medicine, Endocrine Laboratory, Amsterdam UMC Location University of Amsterdam, Academic Medical Center, Meibergdreef 9, 1105AZ Amsterdam, The Netherlands; 3 Amsterdam Gastroenterology, Endocrinology and Metabolism, Amsterdam, The Netherlands; 4 Department of Endocrinology and Metabolism, Amsterdam UMC Location University of Amsterdam, Amsterdam, The Netherlands; 5 Amsterdam Reproduction and Development Research Institute, Amsterdam, The Netherlands

**Keywords:** Graves’ disease, Relapse chance, Prediction model, Antithyroid drugs

## Abstract

**Purpose:**

Graves’ disease (GD) is an auto-immune cause of hyperthyroidism. First-line treatment often consists of a 12–18 month course of antithyroid drugs (ATD). After discontinuation of ATD, GD relapses in approximately 50% of patients. The ‘Graves recurrent event after therapy+ ’ (GREAT+) score may predict individual relapse chances after ATD discontinuation more accurately based on clinical and laboratory parameters at diagnosis. We investigated the need for the GREAT+ score through an online questionnaire among GD patients and physicians treating GD.

**Methods:**

An anonymous online questionnaire was distributed to patients and physicians between June 2022 and August 2023.

**Results:**

The questionnaire was completed by 532 patients and 44 physicians. Results showed that 94% of patients were interested in knowing their GREAT+ score at the start of treatment. 55% would consider definite treatment (radioiodine/thyroidectomy) as first-line treatment in case of a high relapse chance. 98% of the physicians indicated the GREAT + score would support patient counseling. 84% may change their advice for first-line treatment if a patient has a high relapse chance based on the score.

**Conclusion:**

Patients and physicians considered the GREAT+ score as a valuable addition to the current available information which could change treatment decisions. Therefore, external validation of the GREAT+ score is justified to implement this score in clinical practice.

**Supplementary Information:**

The online version contains supplementary material available at 10.1007/s40618-024-02358-7.

## Introduction

Graves’ disease (GD) is an auto-immune disease and the most common cause of hyperthyroidism in iodine-sufficient areas with a prevalence of 0.5–2% [1]. GD is characterized by the presence of autoantibodies that are directed against the thyroid-stimulating hormone (TSH) receptor (TRAb) and thereby able to activate this receptor. This leads to increased free thyroxine (fT4) and free triiodothyronine (fT3) concentrations and suppressed TSH concentrations. TRAbs are often measured to differentiate between GD and other causes of hyperthyroidism, where positive TRAb results (above a pre-defined cut-off value) guide towards GD [2, 3]. Furthermore, TRAbs are measured for follow-up GD treatment [3]. First-line treatment of GD often involves a 12–18 months course of antithyroid drugs (ATD) in the form of ATD titration therapy or combined with levothyroxine (LT4) (block-and-replace therapy). After discontinuation of ATD, GD relapses in approximately 50% of the patients [4, 5]. After a relapse, patients can also choose a more definite form of treatment including radioiodine (RAI) treatment or (total) thyroidectomy, after which life-long LT4 treatment is necessary.

In 2016, the ‘Graves recurrent event after therapy’ (GREAT) and GREAT+ score were developed in the Department of Endocrinology of Amsterdam UMC to improve the prediction of relapse after discontinuation of ATD already at the start of the first-line treatment [6]. These scores were based on four clinical markers: age, fT4 concentration, TRAb concentration, and goiter size. The GREAT + score included the genetic markers tyrosine-protein phosphatase non-receptor type 22 (*PTPN22*) C/T polymorphism and HLA subtypes DQB1*02, DQA1*05 and DRB*03 (Table 1). The GREAT score allocates patients in three different categories corresponding with a relapse chance of 16%, 44% and 68%. The GREAT+ score uses four categories corresponding with a relapse chance of 4%, 21%, 49% and 84% (Table 1). Scores to predict relapse after ATD discontinuation are currently limited to the GREAT( +) score and the clinical severity score (CSS). The CSS score was developed by Bartalena et al. [7] and incorporated fT4 concentration, thyroid volume and the presence of Graves orbitopathy [7].

**Table 1:** Determinants GREAT+ score and GREAT+ categories with relapse chances

Determinants	Score	
Age (years)		
≥ 40	0	
< 40	1	
FT4 (pmol/L)		
< 40	0	
≥ 40	1	
TRAb (IU/L)		
< 6.0	0	
6.0–19.9	1	
≥ 20.0	2	
Goiter size		
Grade 0-I	0	
Grade II-III	2	
*PTPN22*		
Wild type	0	
C/T	1	
HLA polymorphisms		
0	0	
1–2	2	
3	3	

Score GREAT +	Category	Relapse chance (%)
0–2	I	4
3–4	II	21
5–6	III	49
7–10	IV	84

This table is based on the description of the GREAT+ score in the paper of Vos et al. [6].

*FT4* free thyroxine, *TRAb* thyroid-stimulating hormone receptor antibodies, *PTPN22* tyrosine-protein phosphatase non-receptor type 22, *HLA* human leukocyte antigen

The GREAT score has been validated in three independent cohorts [8–10], but the GREAT+ score has not been validated yet. Nevertheless, the addition of a fourth category seems extremely valuable in distinguishing relapse chances, especially in the lowest and the highest categories. Before starting an external validation of the GREAT+ score, we aimed to investigate the need for implementation of the GREAT+ score among patients treated for GD and physicians treating GD.

### Methods

The need for the GREAT+ score was assessed by a questionnaire for current and past GD patients and physicians treating GD patients in the Netherlands. The questionnaire was distributed and completed between June 2022 and August 2023 and supported by the Dutch Thyroid Organization (Schildklier Organisatie Nederland; SON), Dutch Thyroid Research Foundation and the Dutch Society for Endocrinology (NVE) by posting the link to the questionnaire on either their website, social media account, or newsletter. Respondents were not approached personally and could anonymously and voluntarily complete the questionnaire. Therefore, no informed consent from each subject was necessary. Furthermore, the study was not subject to the Medical Research Involving Human Subjects Act since it does not impose any act or mode of behavior on the subjects.

### Patient questionnaire

The patient questionnaire consisted of three sections (Supplementary Table 1). The first section concerned questions on age, sex, and medication (except thyroid medication). The second section concerned 14 questions and dealt with patients’ experience with GD. Before the third section, information regarding the GREAT+ score was provided. After taking note of this information, nine specific questions regarding the GREAT+ score were asked. Closed questions were often followed by open questions.

### Physician questionnaire

The physician questionnaire consisted of two sections (Supplementary Table 2). The first section included four questions regarding experience as a GD treating physician. Before the second section, information regarding the GREAT+ score was provided. After taking note of this information, 14 questions regarding the GREAT+  score were asked. Closed questions were followed by open questions.

### Analysis

The results of both questionnaires were fully anonymous. Questions that subsequently proved redundant were excluded from the analysis. Since most questions were closed, results were merely depicted as a percentage of the total respondents. IBM SPSS Statistics 28.0 (Chicago, IL, USA) and GraphPad Prism 9.3.1 Software were used to analyze the results. Answers from the open questions were used to get insight into the rationale of answers from closed questions, but were not statistically assessed.

## Results

### Patient questionnaire

Table 2 shows the baseline characteristics of the 532 patients who completed the questionnaire. Mean age was 51 years and almost all patients were female (93%). Many patients have had a relapse (48.5%). Interestingly, patients considered GD symptoms as extremely burdensome (mean 4.24 on a 1–5 scale), while side-effects of treatment modalities patients received were considered less burdensome (mean 2.89, 2.73 and 3.56 on a 1–5 scale for, respectively, ATD, RAI and thyroidectomy). A score of 1 indicated no symptoms/side effects; a score of 2 indicated that symptoms/side effects influenced the patients’ life a bit but did not disrupt daily life; 3 indicated that the symptoms/side effects caused some adjustments in daily life; 4 indicated that the symptoms/side effects continuously disrupted daily life; 5 indicated that the symptoms/side effects prevented the individual from carrying out any of their normal daily activities.

**Table 2:** Baseline characteristics of participating patients and physicians

Characteristics	Outcome measure	Outcome
Patients		
Respondents	*N*	532
Age	Mean years (SD)	51 (12.7)
Sex	Female, *n* (%)	496 (93.2)
Relapse after treatment (ATD, RAI, incomplete thyroidectomy)	No, *n* (%)	141 (26.5)
	Yes, *n* (%)	258 (48.5)
	Just started treatment, so not known, *n* (%)	133 (25)
How did you experience the symptoms of GD?	Scale 1–5	Mean 4.24, median 4.0 (*n* = 532)
What GD treatment(s) did you receive?	ATD, *n* (%)	339 (63.7)
	RAI, *n* (%)	24 (4.5)
	Thyroidectomy, *n* (%)	6 (1.1)
	ATD and RAI, *n* (%)	132 (24.8)
	ATD and thyroidectomy, *n* (%)	24 (4.5)
	RAI and thyroidectomy, *n* (%)	1 (0.2)
	ATD, RAI and thyroidectomy, *n* (%)	6 (1.1)
Did you experience side-effects of ATD?	Scale 1–5	Mean 2.89, median 3 (*n* = 380)
Did you experience side-effects of RAI?	Scale 1–5	Mean 2.73, median 2 (*n* = 97)
Did you experience side-effects of thyroidectomy	Scale 1–5	Mean 3.56, median 4 (*n* = 18)
Physicians		
Respondents	*N*	44
Specialized in endocrinology	Yes, *n* (%)	40 (90.9)
Years of experience	Mean	10.1
Number of patients treated for GD per year	Mean	41
Block-and-replace therapy as first line of treatment	*N* (%)	37 (84.1)
Using a tool for indication relapse chance	Yes, *n* (%)	10 (22.7)

*SD* standard deviation, *ATD* antithyroid drug, *RAI* radioactive iodine, *GD* Graves’ disease

**Fig.1:**
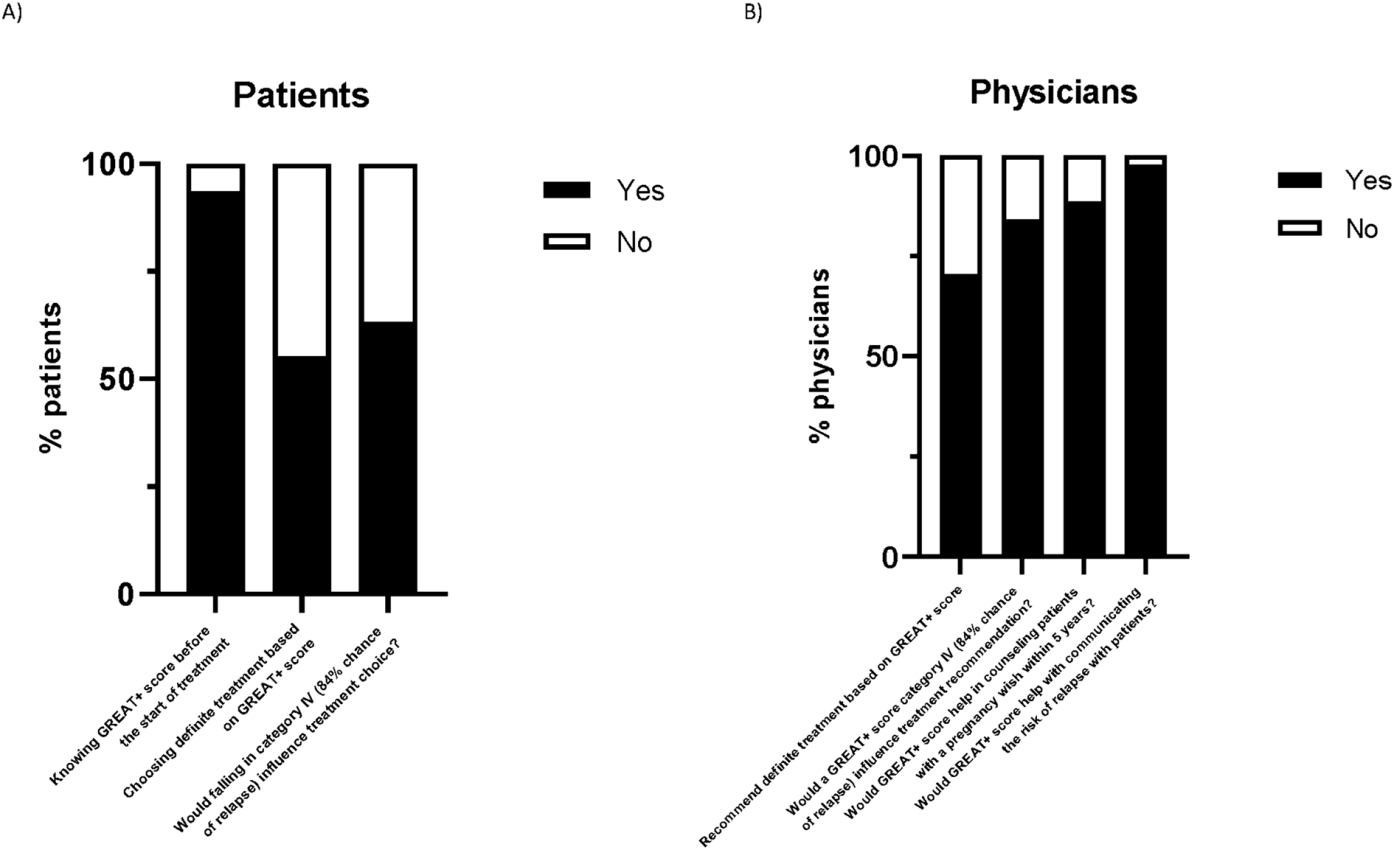
Responses of **A** patients and **B** physicians to GREAT+ score questions

Almost 94% of the respondents wanted to know their GREAT+ score, while a minority (6%) did not (Fig. 1). 55.3% would consider definite treatment based on their GREAT+ result. This even increased up to 63% if their score would fall in category IV (84% chance of relapse).

Open questions revealed several motives for knowing the GREAT+ score. Examples are providing additional information, better expectations about GD relapse, reason for an alternative treatment decision or indication for laboratory testing based on information regarding relapse chance. Patients who did not want to know the score responded it would not change their treatment decision or knowing the score would make them feel less confident during treatment.

### Physician questionnaire

44 physicians completed the questionnaire (Table 2). Ten (22.7%) physicians indicated they already used a tool to predict relapse chance, with four of them specifically using the GREAT score. Figure 1 shows that 70.5% of the physicians reported that the GREAT+ score would make them more likely to recommend definite treatment as first-line therapy, especially if the GREAT+ score would be category IV (84% of the physicians). 88.6% responded affirmatively that the GREAT + score could help them in counseling patients with a pregnancy wish for more suitable treatment. Almost all physicians (97.7%) confirmed that the GREAT+ score would be beneficial in communication with their patients.

The open questions revealed that the GREAT+ score could make physicians better positioned to provide a well-informed recommendation for definite treatment, especially for women in their reproductive age. Other physicians stated they would never start with definite treatment, and the GREAT+ score would not change this. Furthermore, physicians indicated the GREAT+ score could especially be of added value in expectation management and providing personalized information.

## Discussion

The results of our questionnaire showed that both patients and physicians considered the GREAT+ score a valuable addition to the current available information for GD treatment which may change treatment decisions. Therefore, an external validation of the GREAT + score is useful and needed to eventually implement the GREAT+ score in clinical practice.

The majority of patients reported they wanted to know their GREAT+ score at diagnosis. Patients often mentioned uncertainty regarding relapse chance was a large burden, especially since symptoms of active GD were considered more burdensome than side effects of all treatment modalities. Therefore, additional information would contribute to set expectations about the likelihood of relapse. A study from 2019 investigating long-term effects of GD treatment showed that over 25% of GD patients did not feel recovered after a mean follow-up of 8 years [11]. This did not differ per treatment modality and side effects played an important role in this. In our study, we showed a significant burden of side effects for the three treatment modalities, although lower than the burden of GD symptoms. The side effects were rated similarly burdensome for ATD and RAI (median 2.89 and 2.73, respectively), while the burden of side effects for thyroidectomy was higher (mean 3.56). However, this may be caused by the lower number of responding participants with a thyroidectomy (*n* = 18). Previous research evaluated the quality of life of GD patients after receiving either ATD, RAI or thyroidectomy and mostly did not show a difference between the treatment modalities [12, 13], although the quality of life was significantly lower compared to the reference population [12, 14]. Only the study of Törring et al. [14] showed a lower quality of life in RAI-treated patients compared to ATD and surgery [14]. These findings show the large impact of GD before and after treatment.

The GREAT+ score would not change treatment decisions for all patients and open questions indicated that additional information about the disease is a large part of the added value of the GREAT+ score as well. Nevertheless, more than half of the responding patients reported they would more likely opt for definite treatment if they knew their GREAT+ score, especially if the score indicated a relapse chance of 84% (category IV). This indicated the importance of adding this fourth category to the GREAT+ score compared to the GREAT score. A previous study supported this finding by reporting that remission rate was the most important component in treatment decision for GD [15]. Also physicians were interested in the GREAT+ score, and they would advise more likely definite treatment if patients would fall in category IV. Furthermore, physicians reported that results of the GREAT+ score would help in counseling and treating patients with a pregnancy wish within the upcoming five years, which could lead to a different treatment regimen. A European questionnaire from 2010 showed that only 22% of physicians would advise definite therapy for newly diagnosed GD in women with a pregnancy wish, while this increased after a relapse to 80% [16]. Lastly, communicating the risk of GD relapse with patients was found to be a large advantage of the GREAT+ score. A recent study from the US found that in only 3% of their observed consultations with patients with GD an individualized remission estimate was mentioned [17]. The GREAT+ score could, therefore, contribute to firstly discuss this remission or relapse estimate and secondly help to personalize treatment.

The GREAT + score is unique in adding genetic markers which makes it better able to categorize patients into specific relapse chances. These four genetic markers are not included in the standard diagnostic workup of GD and additional costs of measuring these markers in the Netherlands are approximately €300,- per patient. On the other hand, as described by Vos et al. [6] the GREAT + score could lower healthcare costs as well (e.g. better treatment compliance due to additional information provision, and lower treatment costs in case of lower recurrence rate) [6]. Furthermore, it could be debated whether genetic testing should be conducted for all three GREAT score categories or solely for category II. The cost-effectiveness of adding genetics to the GREAT score should thus be evaluated. Moreover, future research should make an effort to further personalize GD relapse chances, for example the use of machine learning could be further explored.

Strengths of this study were the inclusion of a large cohort of patients and the inclusion of physicians as well. A limitation is that we predominantly included patients who were members of the patient association and, therefore, more involved in their disease. Furthermore, the questions were asked retrospectively, so it would be interesting to know how newly diagnosed patients would value the GREAT + score. Our data showed no obvious difference in interest in the GREAT+ score between patients who were in their first-line treatment, patients who experienced a relapse and patients who did not. Patients with a relapse only reported more often that if they would fall in category IV of the GREAT+ score, this would most likely influence their treatment choice (70.2%).

In conclusion, our questionnaire showed that the GREAT + score is of added value for both patients and physicians which justifies the validation and implementation of the GREAT + score in the near future.

## Supplementary Information

Below is the link to the electronic supplementary material.

Supplementary file1 (DOCX 21 KB)

## Data Availability

Raw data may be shared upon reasonable request.
