# Supplementary material for: The need for the GREAT+ score to predict relapse in Graves’ disease: a questionnaire among patients and internal medicine specialists
Source: J Endocrinol Invest. 2024 Mar 25;47(10):2499–505. doi: 10.1007/s40618-024-02358-7 (PMC11393202; doi:10.1007/s40618-024-02358-7)
Supplement: Supplementary file 1 — Supplementary file1 (DOCX 21 KB) [file 40618_2024_2358_MOESM1_ESM.docx]

**Supplementary material**

The need for the GREAT+ score to predict relapse in Graves’ Disease: a questionnaire among patients and internal medicine specialists.

Journal of Endocrinological Investigation

Authors: Heleen I. Jansen, Cecile Heuveling van Beek, Peter H. Bisschop, Annemieke C. Heijboer, Eveline Bruinstroop, Anita Boelen

Corresponding author: Prof. dr. Anita Boelen, Department of Laboratory Medicine, Endocrine Laboratory, Amsterdam UMC, location Academic Medical Center, Meibergdreef 9, 1105AZ, Amsterdam, the Netherlands. Email: a.boelen@amsterdamumc.nl

**Supplementary Table 1.** Patient questionnaire

| **Questions** | **Answer options** |
| --- | --- |
|  |  |
| **Part I: personal information** |  |
|  |  |
| 1. What is your age? |  |
| 1. What is your sex? | Man |
|  | Woman |
|  | Other |
| 1. Are you currently taking any medication not related to Graves' disease? | No |
|  | Yes, namely…. |
|  |  |
| **Part II: Graves’ disease** |  |
|  |  |
| 1. How long ago were you diagnosed with Graves' disease? |  |
|  |  |
| *Information regarding treatment options* |  |
| 1. Has Graves' disease relapsed after treatment with antithyroid drugs, radioactive iodine or incomplete thyroid removal? | Yes |
|  | No |
|  | I have only just started treatment, so I don't know this (yet) |
| *Information regarding remission/treatment* |  |
| 1. Are you currently under treatment or in remission? | In remission |
|  | Under treatment |
| If you are in remission |  |
| 1. On a scale of 1-5 (where 1=not at all and 5=very much), please indicate the extent to which you experienced the symptoms of Graves' disease as burdensome? | 1. No symptoms |
|  | 2. Symptoms influenced your life a bit, but did not disrupt daily life |
|  | 3. Symptoms caused some adjustments in daily life |
|  | 4. Symptoms continuously disrupted daily life |
|  | 5. Symptoms prevented you from carrying out any of your normal daily activities |
| 1. What forms of treatment have you followed? Tick all forms that apply. | Medication (e.g. thiamazole, strumazole, PTU) |
|  | Treatment with radioactive iodine |
|  | Surgical removal of the thyroid gland |
| 1. If you have been taking medication due to Graves' disease, please indicate which medication you have been taking. |  |
| 1. In total, how long have you been treated with medication due to Graves' disease (weeks/months/years)? |  |
| 1. If applicable, please indicate on a scale of 1-5 (where 1=not at all and 5=very much) the extent to which you experienced side effects as a result of the medication. | 1. No side effects |
|  | 2. Side effects influenced your life a bit, but did not disrupt daily life |
|  | 3. Side effects caused some adjustments in daily life |
|  | 4. Side effects continuously disrupted daily life |
|  | 5. Side effects prevented you from carrying out any of your normal daily activities |
| 1. If applicable, please indicate which side effects you experienced as most burdensome. |  |
| 1. Did you have a lot of trouble finding the right dosage of medication? | No |
|  | Yes, in what way… |
| 1. If applicable, please indicate on a scale of 1-5 (where 1=not at all and 5=very much) the extent to which you experienced side effects as a result of radioactive iodine treatment? | 1. No side effects |
|  | 2. Side effects influenced your life a bit, but did not disrupt daily life |
|  | 3. Side effects caused some adjustments in daily life |
|  | 4. Side effects continuously disrupted daily life |
|  | 5. Side effects prevented you from carrying out any of your normal daily activities |
| 1. If applicable, please indicate which side effects you experienced as most burdensome. |  |
| 1. If applicable, please indicate on a scale of 1-5 (where 1=not at all and 5=very much) the extent to which you experienced side effects as a result of the surgical removal of the thyroid gland? | 1. No side effects |
|  | 2. Side effects influenced your life a bit, but did not disrupt daily life |
|  | 3. Side effects caused some adjustments in daily life |
|  | 4. Side effects continuously disrupted daily life |
|  | 5. Side effects prevented you from carrying out any of your normal daily activities |
| 1. If applicable, please indicate which side effects you experienced as most burdensome. |  |
|  |  |
| If you are under treatment |  |
| 1. On a scale of 1-5 (where 1=not at all and 5=very much), please indicate the extent to which you experienced the symptoms of Graves' disease as burdensome? | 1. No symptoms |
|  | 2. Symptoms influenced your life a bit, but did not disrupt daily life |
|  | 3. Symptoms caused some adjustments in daily life |
|  | 4. Symptoms continuously disrupted daily life |
|  | 5. Symptoms prevented you from carrying out any of your normal daily activities |
| 1. What forms of treatment have you followed? Tick all forms that apply. | Medication (e.g. thiamazole, strumazole, PTU) |
|  | Treatment with radioactive iodine |
|  | Surgical removal of the thyroid gland |
| 1. If you have been taking medication due to Graves' disease, please indicate below which medication you have been taking. |  |
| 1. In total, how long have you been treated with medication due to Graves' disease (weeks/months/years)? |  |
| 1. If applicable, please indicate on a scale of 1-5 (where 1=not at all and 5=very much) the extent to which you experienced side effects as a result of the medication. | 1. No side effects |
|  | 2. Side effects influenced your life a bit, but did not disrupt daily life |
|  | 3. Side effects caused some adjustments in daily life |
|  | 4. Side effects continuously disrupted daily life |
|  | 5. Side effects prevented you from carrying out any of your normal daily activities |
| 1. If applicable, please indicate which side effects you experienced as most burdensome. |  |
|  |  |
|  |  |
| 1. If applicable, please indicate on a scale of 1-5 (where 1=not at all and 5=very much) the extent to which you experienced side effects as a result of radioactive iodine treatment? | 1. No side effects |
|  | 2. Side effects influenced your life a bit, but did not disrupt daily life |
|  | 3. Side effects caused some adjustments in daily life |
|  | 4. Side effects continuously disrupted daily life |
|  | 5. Side effects prevented you from carrying out any of your normal daily activities |
| 1. If applicable, please indicate which side effects you experienced as most burdensome. |  |
| 1. If applicable, please indicate on a scale of 1-5 (where 1=not at all and 5=very much) the extent to which you experienced side effects as a result of the surgical removal of the thyroid gland? | 1. No side effects |
|  | 2. Side effects influenced your life a bit, but did not disrupt daily life |
|  | 3. Side effects caused some adjustments in daily life |
|  | 4. Side effects continuously disrupted daily life |
|  | 5. Side effects prevented you from carrying out any of your normal daily activities |
| 1. If applicable, please indicate which side effects you experienced as most burdensome. |  |
|  |  |
| **Part III: GREAT+ score** |  |
|  |  |
| 1. In general, how high do you rate the risk of relapse after stopping medication for Graves' disease (in percentages %)? This question may be difficult to estimate, but it is important for us to know your estimate on this. |  |
|  |  |
| *Information regarding GREAT+ score* |  |
| 1. Would you like to know the GREAT+ score at the start of your treatment? | Yes, because…. |
|  | No, because…. |
| *Repeating information regarding treatment* |  |
| 1. Would knowing the GREAT+ score make you more likely to choose a definite treatment option (radioactive iodine/surgery)? | Yes, because…. |
|  | No, because…. |
|  |  |
|  |  |
| 1. Would you like to know the GREAT+ score independently of your decision regarding first-line treatment? | Yes, because…. |
|  | No, because…. |
|  |  |
| 1. Suppose the GREAT+ score shows that you fall into category I (4% chance of relapse after stopping medication), would that influence your decision on first-line treatment? | Yes, because…. |
|  | No, because…. |
|  |  |
| 1. Suppose the GREAT+ score shows that you fall into category II (21% chance of relapse after stopping medication), would that influence your decision on first-line treatment? | Yes, because…. |
|  | No, because…. |
|  |  |
| 1. Suppose the GREAT+ score shows that you fall into category III (49% chance of relapse after stopping medication), would that influence your decision on first-line treatment? | Yes, because…. |
|  | No, because…. |
|  |  |
| 1. Suppose the GREAT+ score shows that you fall into category IV (84% chance of relapse after stopping medication), would that influence your decision on first-line treatment? | Yes, because…. |
|  | No, because…. |
|  |  |
| 1. Are there any further comments you would like to make about the GREAT+ score? |  |

**Supplementary Table 2.** Physician questionnaire.

| **Questions** | **Answer options** |
| --- | --- |
|  |  |
| **Part I: personal information** |  |
|  |  |
| 1. How long have you been working as an internal medicine specialist (in training)? |  |
| 1. Are you specialized in endocrinology? | Yes |
|  | No |
| 1. How many patients with Graves' disease do you treat per year (estimate)? |  |
| 1. What treatment for Graves' disease do you usually start with? | Thyreostatic drugs, block/replace |
|  | *Which thyreostatic drug do you prefer?* |
|  | Thyreostatic drugs, titration |
|  | *Which thyreostatic drug do you prefer?* |
|  | Radioactive iodine |
|  | Thyroidectomy |
|  | Other, namely…. |
|  |  |
| **Part II: GREAT+ score** |  |
|  |  |
| 1. In general, what do you think the risk of relapse is after discontinuing medication for Graves' disease (in percentages %)? |  |
| 1. Do you currently use any forms of a score or tool to calculate the likelihood of relapse after discontinuation of medication? | No |
|  | Yes, namely…. |
|  |  |
| *Information regarding GREAT+ score* |  |
| 1. Would the results of the GREAT+ score make you more likely to recommend definite treatment (radioactive iodine/surgery)? | Yes, because…. |
|  | No, because…. |
|  |  |
| 1. Suppose the GREAT+ score shows that your patient falls into category I (4% chance of relapse after discontinuing medication), would that influence your advice/decision on first-line treatment? | Yes, because…. |
|  | No, because…. |
|  |  |
| 1. Suppose the GREAT+ score shows that your patient falls into category II (21% chance of relapse after discontinuing medication), would that influence your advice/decision on first-line treatment? | Yes, because…. |
|  | No, because…. |
|  |  |
| 1. Suppose the GREAT+ score shows that your patient falls into category III (49% chance of relapse after discontinuing medication), would that influence your advice/decision on first-line treatment? | Yes, because…. |
|  | No, because…. |
|  |  |
| 1. Suppose the GREAT+ score shows that your patient falls into category IV (84% chance of relapse after discontinuing medication), would that influence your advice/decision on first-line treatment? | Yes, because…. |
|  | No, because…. |
|  |  |
| 1. Do you think the GREAT+ score would change the patient's decision about different treatment options and why? | Yes, because…. |
|  | No, because…. |
|  |  |
| 1. Would you base your treatment advice on the GREAT+ score even if your patient did not want to know their GREAT+ score? | Yes, because…. |
|  | No, because…. |
|  |  |
| 1. Would implementing and discussing the GREAT+ score help you in your communication with patients about the risk of relapse after treatment discontinuation? | Yes, because…. |
|  | No, because…. |
|  |  |
| 1. Would implementing the GREAT+ score help you in communication with a patient with a pregnancy wish within the next five years? | Yes, because…. |
|  | No, because…. |
|  |  |
| 1. Would implementing the GREAT+ score help you in any other way in your patient's treatment? | Yes, because…. |
|  | No, because…. |
|  |  |
| 1. How would you handle the situation if your advice is one way based on the GREAT+ score, but the patient still wishes to be treated differently, where this patient also knows the results of the GREAT+ score? |  |
|  |  |
| 1. Are there any other comments you would like to make about the GREAT+ score? |  |
